# Entering new publication territory in chemoinformatics and chemical information science

**DOI:** 10.12688/f1000research.6101.1

**Published:** 2015-02-04

**Authors:** Jürgen Bajorath

**Affiliations:** 1 Department of Life Science Informatics, B-IT, LIMES Program Unit Chemical Biology and Medicinal Chemistry, Rheinische Friedrich-Wilhelms-Universität, Dahlmannstr. 2, Bonn, D-53113, Germany

**Keywords:** Chemoinformatics, open peer review, chemical structure, active compounds

## Abstract

The *F1000Research* publishing platform offers the opportunity to launch themed article collections as a part of its dynamic publication environment. The idea of article collections is further expanded through the generation of publication channels that focus on specific scientific areas or disciplines. This editorial introduces the *Chemical Information Science *channel of *F1000Research *designed to collate high-quality publications and foster a culture of open peer review. Articles will be selected by guest editor(s) and a group of experts, the channel Editorial Board, and subjected to open peer review.

## Editorial

This editorial introduces a publication channel for the chemoinformatics and chemical information science field. It is primarily addressed to expert investigators who might be interested in exploring new publication territories. Thus, introductory comments will be limited to those required for positioning the *Chemical Information Science* channel. The interested reader who would like to learn more about this research field, its current status, and scientific roots is referred to exemplary reviews ^1– 8^ which provide comprehensive information.

### Chemoinformatics and chemical information science

As a scientific discipline, chemoinformatics has been evolving at interfaces between chemistry, computer science, and information science. When the term chemoinformatics was first coined in the scientific literature ^1^, large pharma companies were primarily in need of chemoinformatics technologies. Hence, there has been a strong -but not exclusive- link to pharmaceutical research. To this date, organization and utilization of drug discovery data involving small molecules, analysis of structure-activity relationships, and prediction of active compounds have remained hallmarks of this field.

Despite its vital link to pharmaceutical research, the development of chemoinformatics can be considered in the broader context of chemical information science ^5^; a view that is also promoted herein. Clearly, chemical structure, data, or text mining and chemical information retrieval for any type of application in the physical or life sciences belong as much to the spectrum of chemoinformatics as computational approaches for the large-scale exploration of pharmaceutically relevant compounds. From this viewpoint, the roots of this discipline go back to the 1950s and 60s, long before chemoinformatics was explicitly discussed.

Given the broad context provided by the chemical information science field, the name *Chemical Information Science* channel was chosen for the new publication platform within *F1000Research*. The *Chemical Information Science* channel is envisioned to cover the wide spectrum of chemical information science (including the chemoinformatics core) and respond to the advent of the *Big Data* era in chemistry ^9, 10^. Some of the issues that have motivated the introduction of the *Chemical Information Science* channel are of general relevance for scientific publications.

### Open peer review

As is the case for many disciplines, there currently is no publication venue available for chemical information science that is based upon open peer review. An open review format will add a new, transparent, and personal quality-control component to the review process that will help to further increase review standards. At least in my opinion, open peer review should become a part of any publication landscape. This aspect in itself provides sufficient justification for adding a missing piece and launching the *Chemical Information Science* channel.

The *F1000Research* platform provides an open discussion forum for any publication, which makes it also possible to comment on reviews, highlight differences in opinion, and trigger further scientific evaluation. This is another important component of open peer review.

### High quality

Publications in many journals are of varying scientific quality, despite peer review. As an example, computational benchmark-type studies that are often not rigorously evaluated and not reproducible represent a recurrent type of publication in the chemoinformatics literature ^11^. Such studies have little value, no potential to advance the field, and will not be considered for publication in the *Chemical Information Science* channel. The same applies to standard applications of molecular modeling software tools.

It is noted that scientific heterogeneity of publications is often a consequence of the need to make journals profitable. At the end of the day, journal issues need to be filled and target numbers reached, which makes it difficult in practice to consistently enforce high quality standards.

Such constraints do not affect the *Chemical Information Science* channel. As an integral part of the operational *F1000Research* publishing platform, the channel is thought to provide an unprecedented opportunity for raising the bar in this field. Simply put, there is no pressure to continuously publish papers. Thus, the quality aspect can be emphasized. The ultimate goal is that each paper appearing in the *Chemical Information Science* channel will be of scientific value to the community – and recognized as such.

### Off-the-beaten-path contributions

According to general scientific publication practices, we are used to exclusively reporting our “success stories”. However, understanding why a computational method does not work or why there might be intrinsic limitations to computational approaches is as important for advancing the field as the development of new concepts and methods. Consistent with the philosophy of *F1000Research*, such off-the-beaten-path contributions are encouraged by the *Chemical Information Science* channel.

### High visibility

The consistent open access provided by *F1000Research* ensures high visibility of publications. The low article processing fees should not provide a substantial barrier for publication in the *Chemical Information Science* channel.

### Editorial board

To help ensure high publication standards in an open peer review setting a group of leading experts will be invited to join guest editor(s) as the channel Editorial Board. Members of the Editorial Board will participate in a two-layer expert evaluation of candidate articles for *Chemical Information Science* channel. Furthermore, interested members will be able to assume periodic guest editorial assignments.

### Two-layer expert review

Submissions to the *Chemical Information Science* channel will be formally evaluated by *F1000Research* editorial staff and then assigned to guest editor(s) who will distribute them to the channel Editorial Board. Board members will initially assess if a submission has the scientific potential to advance the field. Their conclusions will be forwarded to authors. If the initial evaluation is positive, the submission will be published in the *Chemical Information Science* channel and subjected to open peer review. Channel Editorial Board members will help to examine reviewer suggestions of authors and also suggest reviewers. Consistent with principles of open peer review, authors must be in agreement with reviewer assignments. Publications will be indexed once approving reviews are obtained consistent with *F1000Research* standards. A negative initial decision by the channel Editorial Board will preclude publication of a manuscript in the *Chemical Information Science* channel ** (but not in *F1000Research* outside the channel where it can still be subjected to open review). Papers that are published in the *Chemical Information Science* channel and indexed will be designated with a channel label.

### Scope

Apart from general *F1000Research* formatting and data deposition guidelines, there will be no specific rules or thematic restrictions for publications in the *Chemical Information Science* channel. Submissions of all types of articles comprising the current portfolio of *F1000Research* will be considered (except Clinical Practice Articles and Editorials).

### Schedule

The channel infrastructure of *F1000Research* is expected to become operational during the coming months. The *Chemical Information Science* channel will have its own space within the *F1000Research* publishing platform and will be open for submissions in April, 2015.

It will be interesting to see how the chemoinformatics/chemical information science community responds to the addition of an open peer review channel to its publication landscape, with emphasis on high publication standards and fair evaluations. It is hoped that the *Chemical Information Science* channel will be considered as an opportunity to communicate first-class research in an efficient and transparent manner.

## References

[1] Brown FK: Chemoinformatics: What is it and how does it impact drug discovery? *Annu Rep Med Chem.* 1998;33(1):375–384. 10.1016/S0065-7743(08)61100-8

[2] Hann M Green R: Chemoinformatics--a new name for an old problem? *Curr Opin Chem Biol.* 1999;3(4):379–383. 10.1016/S1367-5931(99)80057-x 10419846

[3] Bajorath J: Understanding chemoinformatics: a unifying approach. *Drug Discov Today.* 2004;9(1):13–14. 10.1016/S1359-6446(04)02916-2 14765542

[4] Gasteiger J: Chemoinformatics: a new field with a long tradition. *Anal Bioanal Chem.* 2006;384(1):57–64. 10.1007/s00216-005-0065-y 16177914

[5] Willett P: From chemical documentation to chemoinformatics: 50 years of chemical information science. *J Inf Sci.* 2006;34(4):477–499. 10.1177/0165551507084631

[6] Willett P: Chemoinformatics: a history. *WIRES Comput Mol Sci.* 2011;1(1):46–56. 10.1002/wcms.1

[7] Warr WA: Some Trends in Chem(o)informatics. *Methods Mol Biol.* 2011;672:1–37. 10.1007/978-1-60761-839-3_1 20838963

[8] Vogt M Bajorath J: Chemoinformatics: a view of the field and recent trends in method development. *Bioorg Med Chem.* 2012;20(18):5305–5315. 10.1016/j.bmc.2012.03.030 22483841

[9] Hu Y Bajorath J: Learning from ‘big data’: compounds and targets. *Drug Discov Today.* 2014;19(4):357–360. 10.1016/j.drudis.2014.02.004 24561327

[10] Lusher SL McGuire R van Schaik RC: Data-driven medicinal chemistry in the era of big data. *Drug Discov Today.* 2014;19(7):859–868. 10.1016/j.drudis.2013.12.004 24361338

[11] Walters WP: Modeling, informatics, and the quest for reproducibility. *J Chem Inf Model.* 2013;53(7):1529–1530. 10.1021/ci400197w 23758509

